# Knowledge, attitudes, and practices of oral cancer prevention among students, interns, and faculty members at the college of dentistry of Jazan University

**DOI:** 10.1186/s12903-021-01973-7

**Published:** 2021-12-01

**Authors:** Mosa A. Shubayr, Ahmed M. Bokhari, Afnan A. Essa, Ali M. Nammazi, Dania E. Al Agili

**Affiliations:** 1 Department of Preventive Dental Science, Faculty of Dentistry, Jazan University, Jazan, Saudi Arabia; 2 School of Human Sciences, Faculty of Science, The University of Western Australia, 5 Stirling Highway, Nedlands, Crawley, WA 6009 Australia; 3 Ministry of Health, Jazan, Saudi Arabia; 4 Department of Dental Public Health, Faculty of Dentistry, King Abdulaziz University, Jeddah, Saudi Arabia

**Keywords:** Oral cancer prevention, Knowledge, Attitude, Practice, Dental students, Interns, Faculty members, Jazan

## Abstract

**Background:**

Oral cancer awareness among current and future dental practitioners plays a substantial role in the early detection and prevention of oral cancer. Therefore, this study aimed to assess the knowledge, attitudes, and practices (KAP) of oral cancer prevention (OCP) among oral health practitioners in the College of Dentistry at Jazan University, Saudi Arabia, and to determine factors that facilitate, or limit practices related to oral cancer prevention.

**Methods:**

A self-administered questionnaire survey was done among dental students (n = 274), interns (n = 81), and faculty members (n = 117) in the College of Dentistry at Jazan University between May 2019 to February 2020. The questionnaire was developed in English and modified from a previously validated and published questionnaire into Arabic. It covered every KAP of oral cancer prevention that was useful in accomplishing the study's objectives. Logistic regression analysis was utilized to determine the factors associated with the practice of oral cancer prevention in the past year.

**Results:**

Only 29.7% reported having participated in any OCP activities in the past 12 months while about 42% and 53% of participants referred suspected oral cancer patients to the departments of maxillofacial surgery and oral medicine, respectively. Most of the participants had poor knowledge (71.9%), unfavourable attitudes towards OCP (83.6%) and poor levels of practice (62.9%). The study found that the attitude of the participants was significant in influencing their practices of OCP in the previous 12 months, after adjusting for all other factors.

**Conclusion:**

It was concluded that the level of knowledge, attitudes, and practices of OCP among the sample population was poor. The survey findings suggest that oral health practitioners in Jazan are inexperienced in the methods to adopt for prevention and early detection of oral cancer, despite the high prevalence of oral cancer among province residents. Further research should investigate effective educational strategies and training for improving the participation of students, interns, and faculty members in oral cancer prevention activities.

## Background

Oral cancer is the most common type of cancer in the head and neck region, which develops from the squamous cell epithelium of the lips or oral cavity [1]. Tobacco and alcohol are the most common risk factors for developing oral cancer, and if consumed together, the risk becomes multiplicative [2]. Other risk factors include the presence of chronic inflammation, exposure to ultra violet radiation (in case of lip cancer), genetic predisposition, improper diet, age, oral microbiome such as human papilloma virus (HPV) and immunosuppression [3].

Oral cancers account for more than 354,864 (2.0%) cases and 177,384 (1.9%) deaths worldwide annually [4]. Five-year survival rates depend on the patient's stage of cancer at diagnosis. In the early stages of the disease, the five-year survival rate ranges from 30 to 70% with treatment. Still, in countries with limited medical facilities where the disease is detected much later, this rate may drop lower [5]. In the past two decades, oral cancer has been reported to be on the rise in Saudi Arabia. At present, its prevalence in Saudi Arabia mirrors the international rates of head and neck cancers (HNCs) as the ninth leading cancer [6]. This rise is attributed to the increased use of different kinds of smoked and smokeless tobacco, such as *sheesha* and *shammah* [7, 8]. Jazan province, which is located in south Saudi Arabia has a population of around 1.6 million people [9], and has the highest frequency of oral cancer. It is the most common cancer in female residents and the second most common in male residents [7, 10, 11].

In Saudi Arabia, knowledge and awareness of oral cancer are low among the general public [10, 12]. Up to two-thirds of people in Saudi Arabia are uninformed about the signs and symptoms of oral cancer, and only 12% have heard about oral cancer from their dentists [13, 14]. This lack of awareness is associated with late diagnosis in 50% of oral cancer cases, which leads to the development of advanced stages of the disease and adverse health outcomes [15]. In their study of 679 adults among the general population in Saudi Arabia, Al-Maweri et al. reported that only 53.6% of the participants were aware of oral cavity cancer. Among these, only around one-fifth of them could identify early signs of oral cancer correctly [13].

Oral cancer awareness among current and future dental practitioners plays a substantial role in the early detection and prevention of oral cancer [16, 17]. Some studies noted that dental practitioners do not adequately detect oral cancer at an early stage because of unfavourable attitudes and insufficient knowledge about the symptoms of the disease. Macpherson et al., in a study, found that 58% of dental practitioners conducted regular examinations to detect oral cancer [18] and reported the necessity of conducting more continuing education programs for primary healthcare providers in oral cancer-related activities. Such educational programs can increase knowledge regarding the disease. Most of the surveyed dental practitioners reported a need for additional training diagnosing oral cancer at an early stage to better identify oral malignancies, especially in the early stages [12, 17]. Kujan et al. reported that 87% of surveyed undergraduate dental students in Saudi Arabia felt confident about performing an oral exam to look for signs of such lesions [16]. Therefore, this study aimed to assess oral cancer prevention knowledge, attitudes, and practices among students, interns, and faculty members at the college of dentistry of Jazan University, Saudi Arabia.

## Methods

### Research design

The cross-sectional study aimed to establish a baseline level about the knowledge, attitudes, and practices (KAP) of oral cancer prevention (OCP) among oral health practitioners in the College of Dentistry at Jazan University (JU), Saudi Arabia, and to determine factors that facilitate or limit their practices of oral cancer prevention.

A predefined and validated questionnaire survey was conducted among dental students, interns, and faculty members in the College of Dentistry at JU. Ethical approval was sought from the Institutional Review Board, College of Dentistry, Jazan University. Informed consent was obtained prior to the study.

### Target population

The research project was conducted between May 2019 to February 2020. A list of fifth- and sixth-year dental students, all interns, and total faculty members in the College of Dentistry at JU was obtained from the Vice Deanship for Academic Affairs. A total of 274 undergraduate students, 81 interns, and 117 faculty members were identified as eligible to participate in the survey.

### Procedure

A self-administered questionnaire was distributed in person to all 472 participants. A cover letter about the study information and an informed consent form were distributed and collected back. Participants who agreed to join the study were given the questionnaire and asked to complete it within a week of receipt.

### Study instrument

The questions in the survey were developed after reviewing pertinent literature and modified from a previously validated and published questionnaire [18]. The questionnaire covered every KAP of oral cancer prevention that was useful in accomplishing the study's objectives.

### Arabic translation of the questionnaire

The questionnaire was translated into Arabic by two expert linguists using forward and back-translation. While the first expert translated the questionnaire into English into Arabic (forward translation), the other translated this Arabic version back into English, backward translation by another linguist. Both the original and translated English questions were evaluated and modified by two dental health professors so that the final Arabic version of the questionnaire was formulated. The face validity of the questionnaire was assessed by distributing it to 20 participants and modifying it as needed.

### Reliability and validity

Reliability was assessed using the test–retest method in which 20 participants from the College of Dentistry who were not involved in the study completed the final Arabic version of the questionnaire twice with two weeks intervals in between. The final version of the self-administrated questionnaire included 28 close-ended questions on KAP of oral cancer prevention. Results from the two times were compared using Pearson's correlation coefficient (Pearson's r) as a reliability test. A more significant stability coefficient (Pearson's r) suggested a good test–retest reliability.

### Internal consistency

Internal consistency reflects the inter-correlation between items in the questionnaire and can be quantified using the coefficient alpha "Cronbach's alpha. A Cronbach α = 0.76 was obtained, suggesting adequate internal consistency [19, 20].

### Content validity

Content validity was assessed by distributing this modified questionnaire among the expert panel belonging to the specialty of Oral Medicine. It was rated based on relevance, clarity, simplicity, and ambiguity.

### Scoring

The first part of the questionnaire included information about the demographic characteristics such as age, gender, nationality, education level, and other general questions, while the second part had questions about participants’ knowledge regarding oral cancer prevention. Fifteen statements using "yes" and "no" were used to assess knowledge. It included items such as: "Are tobacco and alcohol the only etiological factors for oral cancer?"; "Do you know the symptoms of oral cancer?"; and "Are early oral cancer lesions always symptomatic?" Every correct response received one point, and so the total knowledge score ranging from 0–15. The cut-off of 9 points represented 60% (9/15) of the total mean of knowledge scores; less than nine represented poor knowledge, and more than 9 represented good knowledge about oral cancer.

In the third part of the questionnaire, respondents were asked about their attitudes toward oral cancer prevention. Participants responded to 7 statements using "yes" and "no" responses. A score of 1 indicated favourable attitudes, while a score of 0 indicated unfavourable attitudes toward oral cancer prevention. The participant's attitude was assessed by using items such as: "I would like more information or training on oral cancer prevention" and "It is a waste of time to educate the patients to quit their habits as they always decline to follow." The maximum score for the attitude scale was 7, with higher scores indicating more favourable attitudes toward oral cancer prevention. The cut-off of 4.2 points represented 60% of the total attitude mean scores; < 4.2 was set as negative attitude and ≥ 4.2 as positive.

The final part of the survey included six questions about oral cancer prevention practices. For example- "Do you examine patients' oral mucosa routinely?"; "Do you record tobacco and alcohol use in the patients' personal history?"; and "Do you practice complete oral cavity examination besides palpating lymph nodes routinely on patients?". The response option was "yes" or "no." The maximum score for this scale was 6, with higher scores indicating a higher frequency of practice. The cut-off of 3.6 represented 60% of the total mean; < 3.6 was set as poor practice and ≥ 3.6 as good practice.

### Data analysis

The questionnaire was pre-coded for entry into the database from a coding sheet. Descriptive statistics (percentages and means) were utilized to provide an overview of each variable. Cross tabulation was formed to examine factors associated with KAP scores. The significance level was set at 0.05. Logistic regression analysis was performed to determine the factors associated with the practice of oral cancer prevention in the past year. Odds ratios and 95% confidence intervals were calculated.

## Results

Out of the 472 questionnaires that were disseminated, 256 questionnaires were completed, which represents a response rate of 54.2% which is represent 61.7% of undergraduate students, 49.4% of interns and 40.2% of the faculty members in the school. The majority of respondents were males (61.7%), ≤ 35 years (83.2%), Saudi nationals (82.0%), and dental students (66.0%). Only 29.7% reported they had participated in any OCP activities in the past 12 months. About 42% and 53% of participants referred suspected oral cancer patients to the departments of maxillofacial surgery and oral medicine, respectively (Table 1).

**Table 1:** Characteristics of study participants (n = 256)

Variables	Frequency	Percentage
Gender		
Male	158	61.7
Female	98	38.3
Age		
≤ 35 years	213	83.2
> 35 years	43	16.8
Nationality		
Saudi	210	82.0
Non-Saudi	46	18.0
Occupation		
Students	169	66.0
Interns	40	15.6
Faculty member	47	18.4
Participation in oral cancer prevention program in the past year		
Yes	79	29.7
No	180	70.3
Referring suspected patients with OC		
Plastic surgery	7	2.7
ENT	4	1.6
Maxillofacial surgeon	108	42.2
Oral medicine	136	53.1
General practitioner	1	0.4

*GP* General Practitioner; *OMFS* Oral and Maxillofacial Surgery; *OC* Oral Cancer

The overall mean (SD) of knowledge about oral cancer among the respondents was 7.66 (1.82). Most (71.9%) of the responding participants had poor knowledge. The overall mean (SD) of attitude towards OCP among the practitioners in the study was 3.44 (1.17). The majority (83.6%) of the responding participants had unfavorable attitudes towards OCP. The overall practice score of OCP among the practitioners in the study was 2.64 (1.28). About two-thirds (62.9%) of the responding participants had poor levels of practice (Table 2).

**Table 2:** Knowledge, attitude and practice of students, interns and faculty members at Jazan University about oral cancer prevention (n = 256)

Variables	Frequency	Percentage	Mean	SD
Knowledge			7.66	1.82
Poor (0–9)	184	71.9		
Good (> 9)	72	28.1		
Attitude			3.44	1.17
Unfavorable (0–4.2)	214	83.6		
Favorable (> 4.2)	42	16.4		
Practice			2.64	1.28
Poor (0–3.6)	161	62.9		
Good (> 3.6)	95	37.1		

*SD* Standard deviation

Table 3 shows the results of the independent t-test and ANOVA, used to determine if there was a significant difference in mean OCP knowledge, attitudes, and practice scores by gender, age, nationality, and occupation of the participants. Only participants’ occupation was significantly associated with the knowledge and attitudes of OCP at the College of Dentistry (p ≤ 0.05). Faculty members (8.40 ± 1.66) had significantly better OCP knowledge scores compared to interns (8.13 ± 1.51) and students (7.34 ± 1.86). Interns (3.78 ± 1.02) and faculty members (3.70 ± 1.02) both showed significant favourable OCP attitudes compared to students (3.29 ± 1.22). The study also found that nationality and occupation were significantly associated with OCP practice in the past 12 months (p < 0.05). Among these, there were 82% of Saudi dental professionals while 18% were non-Saudi. Those who were non-Saudi dental professionals and those who were a faculty member reported good OCP practices (3.33 ± 1.06 and 3.32 ± respectively) compared to Saudi nationals (2.49 ± 1.27), interns (2.93 ± 1.12), and students (2.38 ± 1.29), respectively.

**Table 3:** Knowledge, attitude, and practice of oral cancer prevention among oral health practitioners at Jazan University by gender, age, nationality and occupation (n = 256)

Variable	N(%)	Knowledge		Attitude		Practice	
		Mean(SD)	P value	Mean(SD)	P value	Mean(SD)	P value
Gender							
Male	158 (61.7)	7.27 (1.87)	0.09^a^	3.44(1.22)	0.12^a^	2.60(1.30)	0.33^a^
Female	98(38.3)	8.30 (1.55)		4.43(1.08)		2.70(1.24)	
Age							
≤ 35 years	213(83.2)	7.54 (1.84)	0.33^a^	3.39(1.19)	0.17^a^	2.51(1.27)	0.06^a^
> 35 years	43(16.8)	8.26(1.60)		3.72(1.01)		3.30(1.08)	
Nationality							
Saudi	210(82.0)	7.50(1.82)	0.51^a^	3.38(1.19)	0.23^a^	2.49 (1.27)	**0.03**^**a**^
Non-Saudi	46(18.0)	8.37(1.66)		3.71(1.03)		3.33(1.06)	
Occupation							
Students	169 (66.0)	7.34(1.86)	**0.00**^**b**^	3.29(1.22)	**0.01**^**b**^	2.38(1.29)	**0.00**^**b**^
Interns	40(15.6)	8.13(1.51)		3.78(1.02)		2.93(1.12)	
Faculty member	47(18.4)	8.40 (1.66)		3.70(1.02)		3.32(1.04)	

*SD* Standard deviation

^a^*t*-test of significance

^b^ANOVA test of significance

Logistic regression was conducted to establish the factors that influence the practice of oral cancer prevention in the past year among students, interns, and faculty members in the College of Dentistry at JU (Table 4). The factors that were examined include gender, age, occupation, knowledge, and attitudes toward OCP. Adjusted for all other factors, attitude was the only factor found to be associated with the practice of OCP in the past 12 months. The odds of practicing OCP in the past year was about 20% lower among participants with unfavorable attitudes (OR, 0.80; 95% CI, 0.38–0.69) compared to participants with favorable attitudes.

**Table 4:** Logistic regression model of participation of oral health practitioners at Jazan University in oral cancer prevention activity in the past 12 months (n = 256)

Variables	N (%)		OR	95% C.I
	Yes	No		
Gender				
Male	40 (24.2)	118(46.1)	0.68	(0.38–1.24)
Female	36 (14.1)	62(24.2)	[Reference]	
Age				
≤ 35 years	62(24.2)	151(59.0)	0.51	(0.05–5.66)
> 35 years	14(5.5)	29(11.3)	[Reference]	
Occupation				
Students	37 (14.5)	132(51.6)	1.20	(0.11–12.59)
Intern	24(9.4)	16(6.3)	5.93	(0.53–65.97)
Faculty members	15(5.9)	32(12.5)	[Reference]	
Knowledge				
Poor	61(23.8)	153 (59.8)	0.71	(0.32–1.14)
Good	15(5.9)	27(10.5)	[Reference]	
Attitude				
Unfavorable	27 (10.5)	112(43.8)	0.80	**(0.38–0.69)**
Favorable	49(19.1)	68(26.6)	[Reference]	

*OCP* Oral cancer prevention; *OR* Odd ratio; *CI* Confidence Interval

## Discussion

The purpose of the current quantitative study was to establish a baseline level of knowledge, attitudes, and practices of OCP, and to examine the factors that influence students, interns, and faculty members in the College of Dentistry at JU. The study also investigated if practitioners’ level of knowledge and attitudes toward OCP, when adjusted for other factors, were associated with the practice of OCP in Jazan College of Dentistry.

Based on the descriptive results of the study’s variables, the level of perceived knowledge of oral cancer among the surveyed participants is considered poor. This finding was similar to findings of previous studies by Alsaud [12] in Saudi Arabia and Nazar et al. [20] in Kuwait, but contradicts other studies in India and Saudi Arabia that reported moderate to excellent oral health knowledge among dental students, interns, and faculty members [21, 22]. Additionally, the study found that the level of perceived attitude towards OCP among the practitioners was unfavorable. Our findings contradict the results of studies carried out by Alsaud [12] in Saudi Arabia and Fotedar et al. [22] in India, both of which reported that most participants had a positive attitude toward OCP [12, 23]. Our study also reported that the level of practice of oral cancer prevention was poor. This contradicted the findings from a study from India which reported that many medical and dental practitioners incorporated OCP activities in their practice [22, 23]. It can be speculated that the poor level of oral cancer knowledge and attitudes among the participants in this study in Jazan, Saudi Arabia is the main reason for the poor levels of practice. Finally, our study found that practitioners commonly select oral medicine and oral and maxillofacial surgery as the preferred specialities for referring suspected oral cancer patients, which aligns with the results of previous studies [13, 24].

The knowledge of and attitudes toward oral cancer among oral health professionals in the College of Dentistry at JU were significantly associated with the occupation of study participants. Participants who were working as faculty members had good knowledge scores compared to students and interns. This finding was the opposite of the results found by Jafer et al. [21], who found that there was no difference between students, interns, and faculty members. In their study, interns showed favorable attitudes toward OCP when compared to faculty members or students. This could be because interns might have more clinical time to explore and practice OCP when compared to students and faculty members. In addition, the Saudi Commission for Health Specialities, the licensing association for health care practices in Saudi Arabia, requires that dental graduates participate in several continuous dental education courses in order to apply to any Saudi dental specialty training program.

Occupation and nationality were both significantly associated with the practice of OCP. After adjusting for all study factors, however, attitude toward OCP was the only significant factor in influencing the practice of OCP in the past 12 months. Clearly, participants with less favorable attitudes toward OCP were less likely to participate in OCP activities. The poor knowledge finding in our study was likely to affect participants’ attitudes toward OCP. More continuous education courses and training are recommended to improve the KAP level of early detection of oral cancer among practitioners. The current curriculum of oral cancer prevention in this dental school includes initial assessment, diagnosis and basic treatment provided by the final year students and interns under the supervision of the faculty. In fact, the patients report to them for preliminary treatment and diagnosis.

There were several strengths of this study. This study was a cross-sectional study that allowed researchers to capture a snapshot of the target population regarding the KAP of OCP. Cross-sectional studies provide a quick and inexpensive method of collecting useful information. In addition, this study is particularly useful for informing the planning of education and training of students, interns, and faculty members in OCP, and allocating resources for improving its practice.

On the other hand, some limitations should be considered when utilizing the results of this study. First, our data are cross-sectional and, hence, can be interpreted only as an association rather than a cause-effect relationship. Second, the low response rate of 54.2% is worth noting. The time pressures of day-to-day work may have impeded respondents’ participation in this survey as there was no reminder system. This, combined with a lack of incentives for participation, may have been partly responsible for the low response rate. Although this response rate is low, the rate approximates (56.4%) a study done by Khan et al. [25], which was conducted among dental students and interns in the College of Dentistry, University of Dammam, Saudi Arabia. The small sample size may also have limited the investigation of more variables; however, our study sample is in the same range as other similar studies [21]. Finally, participation in this study was restricted to those who were attending or working at the College of Dentistry of JU, and the limits of the population sampled may limit the generalizability of the results, given the nature and composition of the sample. Therefore, although the results of this study portray an important snapshot of the current situation, they should only be interpreted with caution.

Researchers should investigate effective strategies for improving oral health providers’ participation in oral cancer preventive, educational and training activities. This can be supported by the findings of Nazar et al., who highlighted the need for continuous training on the risk factors and diagnosis of oral cancer so that oral health providers will be able to better understand and apply oral cancer prevention practices in their work. Additionally, our study findings support the need for including oral cancer prevention education and training in dental school curricula in order to improve prevention measures and the treatment outcomes of oral cancer in Saudi Arabia [16]. Clinical dental students should complete several oral cancer screening examinations as part of their training. In addition, the study emphasized the importance of regular training among students, interns, and faculty members to perform oral cancer screenings in their routine examinations in order to practice better oral cancer prevention; increased training would help in reducing the high number of oral cancer cases in Jazan region [26]. Through undergraduate dental education or continued education in oral cancer, trained oral health providers can help in the early detection of oral cancer, hence enhancing oral cancer survival rates and reducing rates of morbidity [27, 28].

## Conclusion

It was concluded that that the current level of KAP is poor relative to the prevalence of oral cancer among oral health professionals in the College of Dentistry at JU. Within the limitations of the study, it can be understood that attitudes of the participants were the only significant factor that influences the practice of OCP. Higher KAP can lead to increased activities and practices of OCP. Further research should investigate effective educational strategies and training for improving the participation of students, interns, and faculty members in oral cancer preventive activities at the Jazan University College of Dentistry.

## Data Availability

All data that support the results of this study are with the corresponding author [M.S.] and can be made available upon reasonable request.
